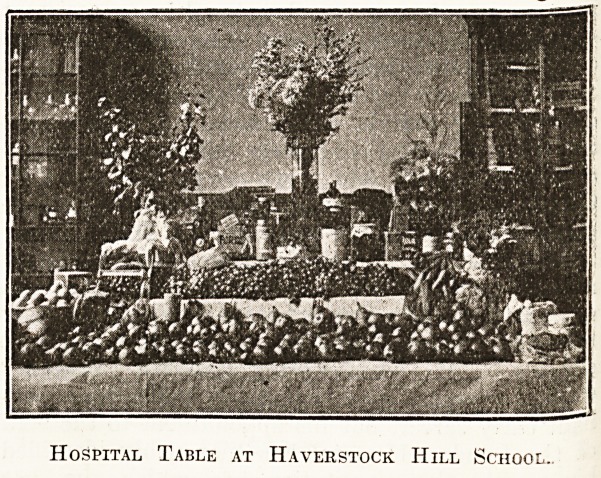# Hospital and Institutional News

**Published:** 1913-01-11

**Authors:** 


### HOSPITAL AND INSTITUTIONAL NEWS.

### THE POSTPONING OF CHRISTMAS FESTIVITIES.

Though every effort was made to include into one number the accounts of Christmas in the Institutions, it is not only or necessarily space which prevents us from doing so. Many institutions, for various reasons, find it convenient to postpone their entertainments, or, perhaps, the most enjoyable feature of them, to the first or second week of the New Year. It would be hard if they should be penalised on that account: for as much energy has been spent, and the result is quite as eagerly looked forward to. Indeed, as this year we have tried to drive home even more persistently than usual, the moral of Christmas in the institutions is that Santa Claus, himself the symbol of personal service for others, is a figure which perambulates the wards of our institutions every day in the year: on Christmas Day he chooses to do so in traditional vestments and with the pomp of ritual. That is all: and that is why Christmas entertainments in institutions lose nothing of the spirit which is lost in other circles of life, and which makes people say: "Do not let us wait till the Christmas spirit has passed." It never passes from the institution, the infirmary, or the hospital that is worthy of the name, and that is why no accounts of the Christmas entertainments are belated in the institutional world.

### THE QUEEN'S HALL DEMONSTRATION.

On Tuesday last the Queen's Hall again witnessed a mass meeting of medical practitioners. This time the tone and temper of the assembly was completely unanimous, and the forcible speeches were echoed to the full. Indeed, the impression received was that of a body of men determinedly and emphatically protesting against scurvy treatment, and fired with the justice of their cause. If there were present any who had signed for one reason or another on to any panel, they could scarcely have been unmoved by the stirring appeals made to them to regard the pledge as still binding on their honour. Scenes of deep enthusiasm followed the brilliant and powerful speech of Mr. E. B. Turner; not mere complimentary appreciation, but the moving enthusiasm of earnest men. Other speakers followed, each almost equally telling in his own way and severely handling the works and promises of Mr. Lloyd George, till when the late Lord Mayor, Sir Thomas Boor Crosby, put the resolution protesting against the unfair methods of intimidation employed by the Chancellor, the whole of the crowded assembly rose literally as one man and carried it. By the irony of fate the most violent speech against the despotism of Mr. George was made by one of his countrymen, who certainly did not mince matters. An interesting attempt of a group of pledged doctors to save their patients from the incursions of the defaulters is given in a succeeding paragraph.

### AN INVITATION TO INSTITUTIONAL WORKERS.

Our readers will have noticed that the Bureau of Information is now included in the Supplement, entitled "The Institutional Worker," to be found every week at the end of the paper. We want all those who are engaged in work in municipal and State institutions, as well as those working for voluntary charities, to aid in making this Supplement of special value. With this in view we are devoting space every week to eliciting facts and solving problems connected with every class of institution for the relief of suffering. Some of the problems will relate to the food supply, prices, preparation, and organisation. Others will relate to methods for checking expenditure; others again to construction difficulties, and to the many complex questions which arise in regard to the admission and classification of patients under the various systems in use. All our readers are invited to cooperate by raising points on which they require information or supplying answers to the problems of others. We hope in this way to have opportunities for contrasting figures collected in various parts of the kingdom and from outside sources. All answers to the questions we have set will receive attention, and such as present interesting features will be published. The payment for each published answer is never less than five shillings. Full particulars will be found on page 2 of "The Institutional Worker" this week.

### THE LONDON PANEL AND ITS COMMITTEE.

The first meeting of the London Medical Committee, which has been elected from the doctors on the panels in London, was held on Tuesday last, The following officials were appointed: Chairman, Dr. J. H. Keay, Greenwich; deputy-chairman, Dr. Claude Taylor, Hampstead; secretaries, Dr. B. A. Richmond, Rotherhithe, and Dr. Welply, Finsbury Park. These officials were also chosen to form part of an executive committee, which consists of fifteen other medical men and women, representing twenty-nine boroughs or districts in the Metropolis.

### MORTON'S FORK IN WILTSHIRE.

The words of Mr. Lloyd George's recent speech, in which he detailed the three turns of the screw by which the medical profession were to be induced to join the panels, are still fresh, we might say sore, in our memories. The "alternatives" were: giving assistants to those already on fairly complete panels; drafting superfluous practitioners to districts where the panels were very incomplete; and the importation of a salaried service—a sort of Morton's fork, only with three prongs, thanks to the ingenuity of a more modern Chancellor. The reply of the British Medical Association refers to "economic pressure, fear of the introduction of outsiders, threats by the Government, and other means, which are perhaps better not further particularised," as the levers which are swaying the hesitating doctors into compliance. As an example to show at least one of the means which is not further particularised above we are informed that it is sufficient for a medical man, who holds a Post-Office appointment and feels his honour binding on him not to deny his pledge, to hesitate to join the local panel to receive a notice at once that unless he does so his appointment will be taken from him. Such deprivation may mean a loss, it is true, of only some £30 a year; but the general practitioner in country districts is dependent very largely on such sums. It would be well worth while for every such case of intimidation to be published, so that it may be placed on record to what extent a political party has been guilty of it. For Mr. H. Samuel's vague disclaimers only add to our disquiet. In the meantime we commend to the notice of those practitioners who are bravely standing by their pledge the manifesto issued last week by a group of Wiltshire doctors, who state the fact of their promise, their powerlessness to break it until formally released, and at the same time their offer to treat insured persons free for a stated period, until some solution be found, and to prevent their own patients from forgetting them. This course seems to us straightforward, and, in fact, the only alternative left to those whose scruples prevent them from denying their pledge. Such men as these were supported by many when the many thought resistance would pay them; and are now deserted the moment that desertion seems profitable. We shall be glad to have our readers' opinions on this policy of Wiltshire medical men.

### DEATH OF THE PRESIDENT OF THE WEST LONDON HOSPITAL.

By the death of the Duke of Abercorn, K.G., the West London Hospital and the Royal United Kingdom Beneficent Association are deprived of their president. In the affairs of the West London the late Duke always took a close personal interest; too close, some people thought, when he exerted his great influence on the board to override recommendations of the medical staff regarding elections of new members to fill vacancies on the latter. Although not an ardent politician, the Duke was immersed in public affairs; especially, of course, in the affairs of the British South Africa Company, as chairman of which gigantic undertaking he succeeded Cecil Rhodes. Yet in spite of his manifold activities he never grudged the time and labour necessary for promoting the interests of the West London Hospital; and his long-continued service to that institution forms an admirable example of the personal service which the voluntary hospital system so frequently evokes. He was elected president eight years ago, and on Christmas Eve visited all the hospital wards in person. On Sunday, last week, he received Queen Amelia of Portugal when she paid a visit to the hospital.

### THE DUKE OF ABERCORN'S FUNERAL.

A Memorial Service for the late Duke of Abercorn was held at St. Mark's, North Audley Street, last Monday, the Sub-Dean of the Chapels Royal and Mr. H. P. Cronshaw, the vicar of St. Mark's, officiating. The West London Hospital was represented at the service by the Secretary, Mr. A. Betteridge, by the Matron, and by several members of the board and of the medical and surgical staffs. The Research Defence Society was represented by Mr. Stephen Paget.

### AN INSURANCE COMMITTEE'S TELEGRAM.

So much has been made of the medical men who have acted contrary to their pledge, that, if only for variety, it is interesting to record at least one instance of compunction on the other side. The Chairman of the Middlesex Insurance Committee, Mr. Glyn-Jones, M.P., has stated that some forty-four Willesden doctors, who had signed the agreement to serve on the panel, had since written or telegraphed asking to be allowed to withdraw. Each of them, he added, had been sent the following telegram: "By signing agreement you have entered into definite contract for three months, from which Insurance Committee absolutely refuse to release you until Committee have made other arrangements in substitution for your service. In no case will the Committee sanction any private arrangements between insured persons and doctors who have broken their contract." If that is not enough to make both insured persons and the medical men to whom it is addressed determined in their course, and inclined, sanction or no sanction, to enter into agreements with each other, the healthy spirit of independence cannot be what it once was. In the meantime counsel's opinion is being taken as to how far the contracts, which the repentant doctors had signed, are legally binding. Such modes of repression and interference between patients and doctors may succeed for the moment: determination to escape from them will revenge itself in the end. That is always the Nemesis of despotism.

### THE INSURANCE ACT AND OUT-PATIENTS: A FORECAST.

"Life would, indeed, be impossible if one had to make a public appeal every year." These words form part of the preface with which the quinquennial appeal on behalf of the London Hospital is sent out. The sum of £100,000 is asked, in addition to the £110,000 which is the figure at which the annual upkeep of the London Hospital is estimated. It is satisfactory to think, therefore, that as its patients came "as a matter of fact from all parts of the globe," so this hospital can cast a wide net, and draws a well-deserved rent of reputation from wealthy and middle-class benevolent persons in every part of the United Kingdom. How will the Insurance Act affect the hospital? "We shall be relieved," writes Mr. Holland, "of some of our out-patients . . . as far as I can calculate; . . . out of 233,500 out-patients seen in a year we shall be relieved of 58,000, and an expenditure on them of about £6,000." That is a very small drop to take out of the ocean of expenditure; and a diminution of one quarter only in the work of the out-patient department, provided always that the wider organisation of ill-health does not bring in new cases to replace those which the Act may take away. All will read with interest this forecast of the effect of the Insurance Act upon a typical large out-patient department.

### STAFF CHANGES IN THE HOSPITALS.

Mindful of the Lister tradition, the Governors of King's College Hospital are not in the least averse from importing distinguished clinicians from outside; the present senior surgeon is an instance in point—namely, Sir Watson Cheyne, who followed Lister from Edinburgh. Now it is announced that, as we detail in the next paragraph, Sir Ronald Ross is to join the staff as physician for tropical diseases, and that in the new hospital at Denmark Hill he is to be allotted beds in which to treat patients thus affected. This can only mean that Sir Ronald, like many another famous man before him, is deserting the provinces for the Metropolis. His work since leaving India has been hitherto identified with the Liverpool School of Tropical Medicine; and it is to be hoped that he will be welcomed gratefully in the London School when he settles in London. Liverpool itself, or rather the Royal Southern Hospital of that city, is advertising vacancies for a gynæcologist and for an assistant surgeon. To the latter post a somewhat unusual condition is attached. The elected candidate must be a Fellow of the Royal College of Surgeons of England, Edinburgh, or Ireland; but, if he is not a fellow of the English College, he must obtain that distinction within four years on pain of forfeiting the appointment. At the London Hospital a surgeon is wanted in the place of Mr. H. P. Dean, M.S., F.R.C.S. The advertisement significantly states that a member of the hospital staff is a candidate for the appointment. This can only mean Mr. James Sherren, the senior assistant surgeon. Mr. Dean was originally a University College Hospital man, where he had a most brilliant early career, but his appointment to the staff of the London Hospital has thoroughly identified him with that institution, to which he has rendered many years of service. Mr. Dean's health has been impaired for some time, and ever since last autumn it has seemed probable that his active connection with the "London" would have to terminate for that reason.

### NEW APPOINTMENT FOR SIR RONALD ROSS.

A foretaste of the opening of the new King's College Hospital on Denmark Hill is the appointment of Major Sir Ronald Ross, F.R.S., as Physician for Tropical Diseases. He will take up his duties in the autumn, when the removal will probably be complete. We need not remind our readers that Sir Ronald Ross is now Professor of Tropical Sanitation in the University of Liverpool and Lecturer on Malaria at the Liverpool School of Tropical Medicine. It is, of course, not unusual for hospitals to give appointments in these subjects, so that any students who desire instruction in these branches of knowledge may have a distinguished teacher at hand. The general interest in tropical diseases has, moreover, increased in recent years, and medical students, as well as the laity, are no less open to impressions such as the recent sanification of the Panama Canal undoubtedly caused. It is probable, therefore, that Sir Ronald Ross's new post will bring him, apart even from personal grounds, a growing amount of work.

### THE "LONG ARM" OF OUR HOSPITALS ABROAD.

We are glad to state that the Council of the Order of the Hospital of St. John of Jerusalem has received a warm tribute from the Viceroy of India, which expresses Lord Hardinge's appreciation of the services rendered by a contingent of the St. John Ambulance Brigade Overseas at the time of the recent bomb-throwing during the State entry into Delhi on December 23. It took humanity many centuries to discover that the arts of peace had as high and practical a function as those of war, and skilled first aid is unique in the sense that the emergencies with which it is studied to deal are those of accident or outrage, which are by no means confined to the battlefield. The Order of St. John of Jerusalem is really the scout or skirmishing arm of the Hospital Service, and connects the voluntary hospitals of this country through the Oversea Brigades with the princes and peoples in many parts of the Empire. The seriousness of the injuries which Lord Hardinge has received must not detract the attention of institutional workers here from the fact that the "long arm" of the hospitals, if one may so express it, has rendered services on the occasion of his attempted assassination, which the Viceroy himself was quick to realise.

### ISOLATION HOSPITALS AND "EARLY CLOSING."

As the result of a discussion between medical men at an inquest lately held at Walthamstow, the need and advisability of performing a tracheotomy at the home of a child suffering from diphtheria—a patient having subsequently died before removal to the isolation hospital—raised the important question whether emergency accommodation should not be provided for such cases. At present it appears to be the recognised custom of the local isolation hospital to receive patients only during the day, so that should a patient be taken with diphtheria at night, and need immediate operation, the medical man is forced to perform the tracheotomy in the private house with all its attendant risks. A medical man in the neighbourhood points out that the public must judge whether it is the duty of the local doctors or of the local authorities to provide a suitable operation room. However small the relative number of emergency cases received may be, this smallness is no excuse for turning them away if they occur at a moment that may not be quite convenient to the authorities of the isolation hospital. For if it be worth while to have an isolation hospital at all it should be worth while to keep it open at night.

### A CORONER'S AMUSING RECOLLECTIONS.

The importance of taking and recording in a book at the hospital the name and address of every outpatient was not the only point of interest which came to light at an inquest last week on a patient of St. Bartholomew's Hospital. The hospital's beadle, Thomas Stow, was in court, and on seeing him the City Coroner, Dr. Waldo, remarked, "I remember you thirty-two years ago," and when some papers found on the deceased were produced by the beadle in the witness-box he added, "*Mist. Rhei*, a favourite remedy at the hospital, is it not? I have given it myself several times when house physician. They are the same old remedies, I notice." A member of the hospital's staff having explained that out-patients were very numerous at Bart.'s, the Coroner said: "In my time we had a number of people attending who were the worse for drink, and we used to have bottles of such and such a mixture labelled 'A, B, C,' or '1, 2, 3.' Is that the custom now?" The answer was a guarded affirmative! The Coroner told the jury that the Insurance Act might reduce the number of out-patients, and thus enable the hospital authorities to register the name and address of every patient received. It is an open question, perhaps, whether the jury were as much impressed with the Coroner's forecast as they were amused at his recollections of a house physician's experience in a large out-patient department at a London hospital.

### DEATH OF AN INSTITUTIONAL ARCHITECT.

The death is announced of Mr. Francis James Smith, F.R.I.B.A., in his sixty-eighth year. As architect to the Shoreditch Guardians he carried out the Hornchurch Cottage Homes and the Nurses' Home at Shoreditch Workhouse, and as architect to Paddington Guardians he carried out extensive works, comprising the new board room and offices, receiving and casual wards, and the reconstruction of the Harrow Road Workhouse, also the Nurses' Home and Dispensary buildings. As architect to the St. George's, Hanover Square, Guardians, the St. Leonard's, Shoreditch, Board, and the Bromley Union Board he also carried out extensive alterations and improvements. We are glad to reprint this record, for an immense amount of scattered but useful and exacting work is carried out by numbers of hospital architects, whom chance or disposition has never thrown in the way of imposing schemes, but who do their share in institutional progress. The big scheme may be a record of fad or fashion, the small scheme is often the best indication of a growing consciousness of real progress, for limited capital may mean that only accepted improvements are embodied.

### THE ORDER OF MERCY.

The fact published in The Hospital of last week that the League of Mercy has contributed a total amount of £200,000 to the voluntary hospitals lends special interest to the awards which the King, as Sovereign of the Order of Mercy, has sanctioned to various lady presidents, vice-presidents, lady vice-presidents, and members of the League. The full list of recipients will be found in another column. Here we would only say that the sum-total which ten years of personal service on the part of small givers and collectors has been able to aggregate shows, as anyone who understands the system of collection will realise, that the Order of Mercy is a true symbol of that giving of themselves to others, which has brought through the League a meaning into many lives. The narrowness of riches and poverty alike yield to the League's secret, which has brought something of the institutional spirit into the lives of those outside its direct influence.

### SCHOOLBOYS AND A LONDON HOSPITAL.

Haverstock Central, N.W., is one of the most interesting of the higher-grade London County Council schools in London, not only because as a training centre it possesses certain almost unique features, but also because of the practical interest which the pupils take in the hospitals in their neighbourhood. The school possesses its own magazine, which is edited, printed, and published by the boys; it also has a special class for enamel work, and has lately started a photography class. The accompanying illustration is made from a print of a negative taken by a member of the class, and represents the hospital table, which this term was a record one.

Hospital Table at Haverstock Hill School.

The Headmaster, Mr. F. R. Barnes, to whose personal effort and initiative much of this special school activity is due, is a warm friend of the voluntary hospitals, and it has been his custom at the various schools of which he has been Principal to stimulate the pupils' interest in these institutions by holding an annual "Hospital Day." This, he explains, is quite a voluntary affair on the children's part. All that authority does is to give a short talk to the school at morning assembly, pointing out how those who are in health may think of those who are sick and suffering. The text of this little sermon is the stanza:

> We can all do more than we have done
> And not be a jot the worse;
> It never was loving that emptied the heart,
> Nor giving that emptied the purse.

On "Hospital Day" the pupils bring various little gifts, and the nature of the response is best seen from the items that figure on the hospital table. Among these may be noticed new-laid eggs, cocoa, tea, sugar, and other provisions, fresh fruit, wine, honey, flowers, and magazines and books. Each boy lays his contribution on the table which is arranged by one of the teaching staff, and after the whole school has viewed the collection each class elects a boy as its representative to take the tribute to the hospital selected. The school supplies a cab, and the deputation proceeds to the hospital—in this case the Hampstead General Hospital—where the boys hand their gifts to the Matron. The little outing is a practical lesson for all concerned, and we trust that the Hospital Secretary is alive to the value of the educational interest attaching to such a visit. We suggest that it would not be a bad policy for the authorities of the Hampstead Hospital to emulate the initiative of the West Ham Hospital Chairman and invite a few representatives from the schools in the neighbourhood to visit the institution and see for themselves what a modern hospital is like. Such a visit will undoubtedly be stimulative and may win the hospital new friends and supporters. Meanwhile, we congratulate Mr. Barnes and his boys on the excellent success of their "Hospital Day" this year.

### WHAT IS AN HONORARY OFFICER?

The Wells Cottage Hospital, which is older than might appear from the recently stated number of its annual meetings, has decided that all honorary officers shall be considered as subscribers, and as such shall be entitled to vote at all general meetings. It becomes important to inquire, therefore, what is meant by the term "honorary officer." A dictionary definition would no doubt describe such a person as an officer who receives no emolument. On the other hand, an honorary officer has been defined, often quite truly, as an officer who receives an honorarium. For there is a real and practical distinction between salary or emolument and honorarium; the latter word has been invented for a purpose of its own. For instance, a secretary may be engaged for an institution at a certain salary, in fact this is the rule. But there is an alternative to not paying him—namely, to give him to understand that the board or some other body will almost certainly give him a present at the end of the year—say, fifty guineas—but that such a gift is no part of the actual contract. An honorarium is a present, not a wage. None the less, it is important to bear the two meanings in mind where such a rule is passed as this at the Wells Cottage Hospital. If honorary means "without a salary" and "without a present," the decision takes a different complexion from that which would attach to it if honorariums are being paid to the officers implicated in the change.

### THE BEQUEST OF AN ECCENTRIC DOCTOR.

Rather more than a year ago, to be precise on December 23, 1911, we began a paragraph about the proposed Royton Hospital, near Oldham, with this statement: "If you want to revenge yourself upon posterity perhaps the best way, as experience teaches, is to leave money for the foundation of a new institution or for the endowment of a new need." A year later all but three days the statement is published that "it is over three years since the doctor (Dr. Kershaw) died, and nothing material seems to have been achieved yet." Dr. Kershaw, whose eccentricities endeared him to his friends, left £40,000 and plans for an institution of thirty beds, which he projected at a cost of £11,000, leaving the remainder for endowment purposes. The District Council, however, fearing that it would have to provide an additional income of £1,100 to maintain an institution of this size, handed the bequest over for consideration to a sub-committee. The result, of course, was that a cottage hospital was projected last spring, in the hope no doubt that the sum left over from building a smaller institution would be sufficient to maintain it without much or any voluntary help. The trustees seem to have had the matter in their hands for many months, but the architect has been chosen—Mr. Sydney Moss, of Manchester, who has been working on the plans. It is expected that building will begin in March, the site of the much debated hospital being on Dr. Kershaw's estate in Turf Lane, Royton. What is the attitude of Oldham Infirmary to this proposed daughter institution and to its delayed establishment?

### BIG GAME AS A MEDIUM OF INFECTION.

In a lecture on "Sleeping Sickness and Big Game" Dr. Warrington Yorke, director of the Runcorn Research Laboratories of the Liverpool School of Tropical Medicine, stated that the tsetse fly, which was known to cause "fly disease" in domestic stock, *Glossina morsitans*, has been proved to be the cause of sleeping sickness in man. The main reservoir of the trypanosomes of man and domestic animals, he added, is the big game. In answer to the question which at once presents itself: What, then, can be done to stamp out or control sleeping sickness in Nyassaland and Rhodesia? Dr. Yorke says, in effect, we cannot at present get rid of the fly. Moreover, as the big game rather than the infected person is the reservoir of the virus, the infected person is not the source to isolate but the big game. All proposals for dealing with big game have excited great controversy, and therefore Dr. Yorke advocates not their destruction, but their driving back from the neighbourhood of human habitation. He recommended that an experiment on a sufficiently large scale should be scientifically carried out on lines that he elaborated. It must be remembered that this is not a question for sportsmen alone.

### MEDICINE AS AN EDUCATION.

The President of the Board of Education has appointed an Advisory Council for the Science Museum to offer opinions on questions of principle or policy, and further to make an annual report of its proceedings to the Board, together with any observations as to the condition and needs of the Museum which they may see fit to make. The first members include the following medical men: Sir Alfred Keogh, K.C.B., LL.D., M.D., F.R.C.S.I., and Sir William Ramsay, K.C.B., LL.D., D.Sc., M.D. The Secretary will be Captain

H. G. Lyons, F.R.S., of the Science Museum. That the well-known scientists, of whom the Advisory Council consists, include two medical men is interesting as an example of the high value which medicine has as an education in the best sense of the word. It is so often asked what studies can take the place of classics in education that the value of medicine in this respect alone is worth emphasising. Moreover, it provides its students with a reasonable opportunity of earning a living at the close of their studies, such as only the two professions of parish priest and schoolmaster give to those whose education resolves itself mainly into a study of Latin and Greek.

### THE LEASE OF A LONDON EYE HOSPITAL.

It is announced that the present premises of the Central London Ophthalmic Hospital, at the corner of Calthorpe Street and Gray's Inn Road, are to be let on lease, in view of the approaching transfer of the hospital to premises in Judd Street. It would be idle to pretend that the Calthorpe Street-Gray's Inn Road buildings are dignified or hospital-like in appearance. In fact they are dull and dingy to a degree. But that need not necessarily be any detriment to their acquisition for commercial purposes; and for the sake of the work which the hospital does, we hope they will speedily find a tenant at a good rental. A rival institution, the Royal London Ophthalmic Hospital (still known to most people by its old title of affection—"Moorfields"), has been presented, by the way, by one of its consulting surgeons, Mr. William Lang, F.R.C.S., with £450 to endow a special clinical research scholarship for three years.

### INSURANCE ACT AND CHEMISTS' PANELS.

Chemists' panels have now been formed in all districts in Great Britain, the majority of the chemists in each area having signified their willingness to supply drugs and appliances in accordance with the terms of the National Insurance Act and the regulations as to medical benefits. In many instances the Insurance Committees have already made agreements with the local pharmaceutical associations with regard to the prices of drugs and appliances and charges for dispensing. The prices arranged by the Committees are based on a tariff which was drawn up by the Pharmaceutical Standing Committee on Insurance, and in some cases the Committees have agreed to allow extra charges for medicines supplied at night, subject to the approval of the Insurance Commissioners.

### THE INSTITUTIONAL WORKERS' READY REFERENCE.

The new 1913 "*Nursing Mirror* Pocket Encyclopædia and Diary," published by The Scientific Press, Ltd., 28 and 29 Southampton Street, Strand, W.C., is a wonderful compendium. From "How to Make Beds" to "Notes on Chiropody" it contains in a few terse, and where necessary illustrated, paragraphs all information required at the threshold of the professions in institutional life. The would-be dispenser, nurse, or midwife, for instance, find here a summary of the chief points likely to be useful to them from a whole array of text-books and professional guides. As a sixpenny ready reference for the institutional worker generally it is, seriously, unrivalled, and no better collection of aphorisms on the hospital spirit and on nursing can be found than its quotations from Florence Nightingale's "Maxims," which the courtesy of Messrs. Harrison and Co. has allowed the proprietors to publish. We cordially commend the volume to every class of institutional worker.

### A CLIQUE OF INJUDICIOUS EXAMINERS.

There is a certain amount of dissatisfaction being expressed in London surgical circles over the manner in which the examinations for the degree of Master in Surgery (M.S.) are conducted. This degree has a very high standing indeed: higher in surgery even than the corresponding degree has in medicine (M.D.). It is alleged by malcontents: first, that this disparity was never intended when the degrees were instituted; and, secondly, that the standard is being further raised to an artificial height by a policy of wholesale rejections. It appears to be the case that at the last examination for the M.S. twelve out of fifteen were "ploughed," and the previous time twelve out of fourteen suffered the same ignominious fate. When it is stated that the candidates are almost without exception Fellows of the Royal College of Surgeons, and devoting themselves to surgery as a specialty, it will be realised how very stringent the examination is being made. The case for the University has not been stated; but, unless it traverses the facts set out above, we cannot imagine that justification of the policy now being followed can be maintained. We prefer to think that a clique of injudicious examiners have been allowed to place the University in an awkward position, from which a speedy retreat is the only sound strategy.

### THIS WEEK'S DRUG MARKET.

Business in the Drug market is beginning to brighten up, and, although at present the demand is confined to a few articles only, the outlook is distinctly hopeful. A large business has been done in quinine, but there has as yet been no advance in the prices quoted by the makers; the demand has no doubt been stimulated by the rumours that negotiations with regard to restricting the output of bark, between the planters of cinchona bark in Java and the manufacturers of quinine, are nearing completion; but it remains to be seen whether these rumours have any solid foundation. The importers of quicksilver have announced an advance in price, and it is not improbable that makers of calomel, corrosive sublimate, and other salts of mercury may increase their prices accordingly. Cloves continue to fetch high prices, and the value of oil of cloves is tending in an upward direction. An advance in the price of bromides at an early date is considered probable. Essence of lemon continues to rise in price, and the value of sandalwood oil seems likely to advance still further. The high price of olive oil is well maintained, with an upward tendency.

## Figures and Tables

**Figure f1:**